# Graph literacy matters: Examining the association between graph literacy, health literacy, and numeracy in a Medicaid eligible population

**DOI:** 10.1371/journal.pone.0241844

**Published:** 2020-11-11

**Authors:** Marie-Anne Durand, Renata W. Yen, James O’Malley, Glyn Elwyn, Julien Mancini

**Affiliations:** 1 The Dartmouth Institute for Health Policy & Clinical Practice, Dartmouth College, Lebanon, NH, United States of America; 2 UMR 1027, Université Toulouse III Paul Sabatier, Toulouse, France; 3 Aix-Marseille Univ, APHM, INSERM, IRD, SESSTIM, “Cancer, Biomedicine & Society” Group, Hop Timone, Marseille, France; University College London, UNITED KINGDOM

## Abstract

**Objectives:**

Graphic display formats are often used to enhance health information. Yet limited attention has been paid to graph literacy in people of lower education and lower socioeconomic status (SES). This study aimed to: 1) examine the relationship between graph literacy, numeracy, health literacy and sociodemographic characteristics in a Medicaid-eligible population 2) determine the impact of graph literacy on comprehension and preference for different visual formats.

**Methods:**

We conducted a cross-sectional online survey among people in the US on Medicaid, and of presumed lower education and SES.

**Results:**

The mean graph literacy score among 436 participants was 1.47 (SD 1.05, range: 0 to 4). Only graph literacy was significantly associated with overall comprehension (p < .001). Mean comprehension scores were highest for the table format (1.91), closely followed by bar graph (1.85) and icon array (1.80). Information comprehension was aligned with preference scores.

**Conclusions:**

Graph literacy in a Medicaid-eligible population was lower than previous estimates in the US. Tables were better understood, with icon arrays yielding the lowest score. Preferences aligned with comprehension.

**Practice implications:**

It may be necessary to reconsider the use of graphic display formats when designing information for people with lower educational levels. Further research is needed.

## Introduction

Understanding health information and related numerical information is critical to making informed decisions, promoting adherence to treatment, and improving health outcomes [1–8]. It is estimated that about 30% of the general population in the United States (US) has limited numeracy [9]. Limited numeracy is widespread and varies significantly between OECD (Organization for Economic Co-operation and Development) countries, with the US ranked 29 out of 35 countries on numeracy skills, considerably below the OECD average [10]. In people of lower educational attainment, processing numerical information is even more difficult than for more educated groups [11]. In other words, there is a gap in numeracy skills between people of lower and higher educational attainment [11]. Numerical health information can be presented as numbers only or graphically, using graphic display formats [12]. Graphic display formats, such as pie charts, bar charts, line plots, and icon arrays are frequently used to enhance communication [13–15]. They can minimize denominator neglect [16], framing effects [17, 18], and the effect of anecdotal reasoning [19]. Graphic display formats can also lead people to overestimate low probabilities and underestimate high probabilities [20]. Few studies have explored risk communication and the ability to understand graphic displays of risks in people of lower educational attainment and lower socioeconomic status (SES) [21].

While pictures may be worth a thousand words, a graph is more complex than a picture [22]. Extracting and understanding information presented graphically requires a specific set of skills, called graph literacy, also known as graphicacy [22–25]. Graph literacy is a concept that has so far received limited attention [23, 26]. Bar charts, pie charts, and line plots were first introduced in the late 18^th^ century [24, 27], with icon arrays later appearing in the early 20^th^ century. Galesic and Garcia-Retamero suggest that there is therefore no obvious reason why people should intuitively understand information presented graphically [23]. Graph literacy requires the ability to extract information from two-dimensional images, to read data, and compare the information of interest to other groups or categories of information [24]. Graphic display formats may not benefit all adults in the same way. People with lower graph literacy may not always benefit from graphic displays of information, and may process information more accurately with numbers alone [22, 23, 28]. Our study of the acceptability and feasibility of patient decision aids among women of lower SES suggested that many participants struggled to comprehend numerical estimates of risk presented as icon arrays. In the pictorial patient decision aid group, about 77% of women of lower SES who completed the online survey found icon arrays confusing [29].

Galesic and Garcia-Retamero have investigated the level of graph literacy in the US and Germany [23]. In both countries, about one third of the population had low graph literacy. Nayak et al. assessed graph literacy skills in a highly educated sample of prostate cancer patients (78% college educated). Graph literacy and numeracy were positively correlated, suggesting that people with limited numeracy also have limited graph literacy. Despite high educational attainment and high health literacy levels, considerable variations in the ability to understand graphs were observed [22]. Graph literacy scores were more strongly correlated with dashboard comprehension scores than numeracy scores. If graph literacy was the strongest predictor of dashboard comprehension scores in Nayak’s highly educated sample, this tendency is likely to be exacerbated in people of lower educational attainment. However, as far as can be determined, no studies to date have assessed graph literacy in people of lower educational attainment and lower SES. This study thus aimed to: 1) examine the relationship between graph literacy, numeracy, health literacy and sociodemographic characteristics in people of presumed lower SES and 2) determine the impact of graph literacy on comprehension and preference for different visual formats as well as the relationship between comprehension and preference. The results will be useful in tailoring medical information to people of lower SES and presumed lower numeracy and lower graph literacy in the most effective way to enhance understanding, thus following a proportionate universalism approach [30].

## Methods

We conducted a cross-sectional online survey using a survey sampling panel on Qualtrics. The study was designed, conducted, and reported according to CHERRIES (CHEcklist for Reporting Results of Internet E-Surveys) (S1 File) [31].

### Participants

We used self-reported current Medicaid enrollment as a proxy measure of lower SES to recruit people of presumed lower SES and lower educational attainment living in the United States (US) (determined using panel IDs). Medicaid is a joint federal and state program that provides health coverage to over 72 million people in the US. Medicaid enrollees include low-income families, qualified pregnant women and children, individual with disabilities and individuals receiving Supplemental Security Income (SSI). We asked the Qualtrics team to target the recruitment of Medicaid enrollees on their panels. In addition, we used three screening questions aiming to only include participants who reporting being at least 18 years old, being a Medicaid enrollee, and felt comfortable reading and completing a survey in English. We based the sample size on the estimated number of adults in the US enrolled in Medicaid and using a 95% confidence interval with a 5% margin of error. We identified unique visitors using unique panel IDs and checked for duplicates. Qualtrics does not use IP addresses to check for duplicates. In addition, cookies were placed upon entering the survey to prevent participants from completing the survey more than once.

### Survey administration

The survey was exclusively open to Qualtrics’ active panel participants. Qualtrics (www.qualtrics.com) is a customer experience company and online survey platform that sends online surveys to a targeted population of respondents. A survey invitation containing a link was sent out to them based on the profiling data provided to Qualtrics (described above). To encourage participation, the participants were offered points that they could redeem for prizes.

### Survey design

The survey had 13 pages (S2 File). Except for the page that provided study information, consent, and instructions, each page contained between zero and six questions. Each page included a progress bar but no back button. The survey had a total of 34 questions including the consent and screening questions (see Table 1).

**Table 1:** Overview of survey structure.

Page number	Section	Number of Questions
1	Introduction and consent	1
2	Screener 1	1
3	Screener 2	1
4	Screener 3	1
5	Demographics	6
5	Health literacy	1
6	Graph literacy	4
7	Subjective numeracy	3
8	Introduction to comprehension section	0
9	Comprehension section 1*	5
10	Comprehension section 2*	5
11	Comprehension section 3*	5
12	Preferred visual format	1

* The order of the three visual formats was randomized.

An information sheet and consent form appeared on the first page of the survey. It briefly described the purpose of the study, what participation would involve, and stated that participants may opt out at any time. The next three pages had one screening question each to check that the participant met the inclusion criteria of being: 1) over the age of 18, 2) currently enrolled in Medicaid, and 3) comfortable completing the survey in English. Participants were subsequently asked to provide demographic information (six questions) and answer the one-item health literacy question. The next two pages tested graph literacy (four questions) and subjective numeracy (three questions). The following page provided a short introduction to the comprehension questions. The subsequent three pages each assessed comprehension of fictitious risk information using icon arrays, bar graph, and table (three questions each). We purposefully chose not to use pie charts as previous studies have demonstrated that this format leads to slower and less accurate responses [15, 32]. The order of the three visual formats was randomized to check for interaction effects with other study variables. The last page asked participants to indicate the visual format they found most helpful in presenting risk information (one question). No personal information that could link participants back to their identity was collected.

#### Health literacy

We measured health literacy using Chew’s one-item scale, “How confident are you in filling out medical forms by yourself?” The answer choices were extremely, quite a bit, somewhat, a little bit, and not at all [33]. Based on the validation study, those who answered extremely and quite a bit were assigned to the adequate health literacy group; the rest were assigned to the limited health literacy group, which included both inadequate and marginal health literacy. This was treated as a dichotomous variable.

#### Subjective numeracy

We measured subjective numeracy using the validated 3-item Subjective Numeracy Scale (SNS3), which asks respondents to rate their perceived ability in using numbers (S2 File) [34]. The three items are scored on a scale of 1–6 (1 = Not at all good or never to 6 = Extremely good or very often) and the sum of three answers is the SNS3 score (range 3–18) [34–37]. For analysis, we created a dichotomous variable. Participants with scores above the median score were assigned to the “higher” SNS3 group while those with scores lower than the median score were assigned to the “lower” SNS3 group.

#### Graph literacy

To minimize burden on respondents, we used the validated 4-item short version of the graph literacy scale [26]. The graph literacy scale asks participants to interpret four different graphs with one question per graph (S2 File). Each participant was given a graph literacy score based on the sum score of four questions (range 0–4). We created a dichotomous variable of high and low graph literacy group based on whether the score was above or below the median score. Participants with graph literacy scores above the median score were assigned to the “higher” graph literacy group while those with scores lower than the median were assigned to the “lower” graph literacy group.

#### Comprehension

Participants viewed hypothetical recurrence risks of cancer treatments in three different formats: table, bar graph, and icon array. They answered three comprehension questions following each visual display format. The first question required basic interpretation of the recurrence risk after one treatment option (gist task); the second required expressing the recurrence risk out of 100, 10 years after a given treatment (verbatim task), and the third question required comparing the difference in recurrence risk between two options (verbatim task). Each correct answer was given a point of 1, each incorrect answer was given a score of 0. Answers to free text questions were dual coded with conflicts discussed with a third coder. The comprehension score for each format equaled the number of first three questions answered correctly (range 0–3). We also examined the total comprehension score, which was the sum of the scores for all formats (range 0–9).

#### Preference

For each visual display format (table, bar chart, icon array), we asked about overall preference for visual display format.

### Survey development

The survey was developed in Qualtrics using the validated scales described above. Other items (such as the comprehension questions) were developed by the research team, based on existing literature in this area, and tested with a convenience sample of research collaborators and lay users. Testing focused on the usability and readability of the written content of the survey and visual display formats embedded in the survey. Only minor formatting and typographical edits were made.

### Statistical analysis

#### Analyses corresponding to our primary aim

We performed simple logistic regressions to assess the unadjusted relationship between sociodemographic factors including gender, education, having one or more chronic conditions, and having family history of cancer and the dichotomous variables for health literacy, SNS3 and graph literacy. We also performed a multiple logistic regression with graph literacy as the dependent variable and all sociodemographic variables, SNS3, and health literacy as independent variables to assess how each independent variable was associated with graph literacy.

#### Analyses corresponding to our secondary aim

We performed one-sample t-tests to determine if there were differences in comprehension scores by format. We performed simple logistic regressions to assess the unadjusted relationship between graph literacy and overall comprehension and graph literacy and preference. We performed a multiple logistic regression to evaluate whether the relationship between graph literacy and comprehension changed when controlling for preference, SNS3, health literacy, age, gender, education, having one or more chronic conditions, and having family history of cancer with graph literacy as the dependent variable and all other factors as independent variables.

We performed simple linear regression to assess the relationship between preference and overall comprehension score. We performed multiple linear regression to assess this relationship while controlling for SNS3, health literacy, age, gender, education, having chronic conditions, and having family history of cancer.

#### Assessing for order effects

We performed a simple linear regression to assess if overall comprehension score was different based on which graph format was seen first. We also looked at the individual format scores based on format order using simple logistic regressions to determine if individual format scores varied based on which format was seen first. A logistic regression was used since we dichotomized the variable of icons seen first (yes/no as dependent variable).

The study was approved by the Committee for the Protection of Human Subjects (CPHS) at Dartmouth College on August 7, 2017.

## Results

### Participants

**Fig 1:**
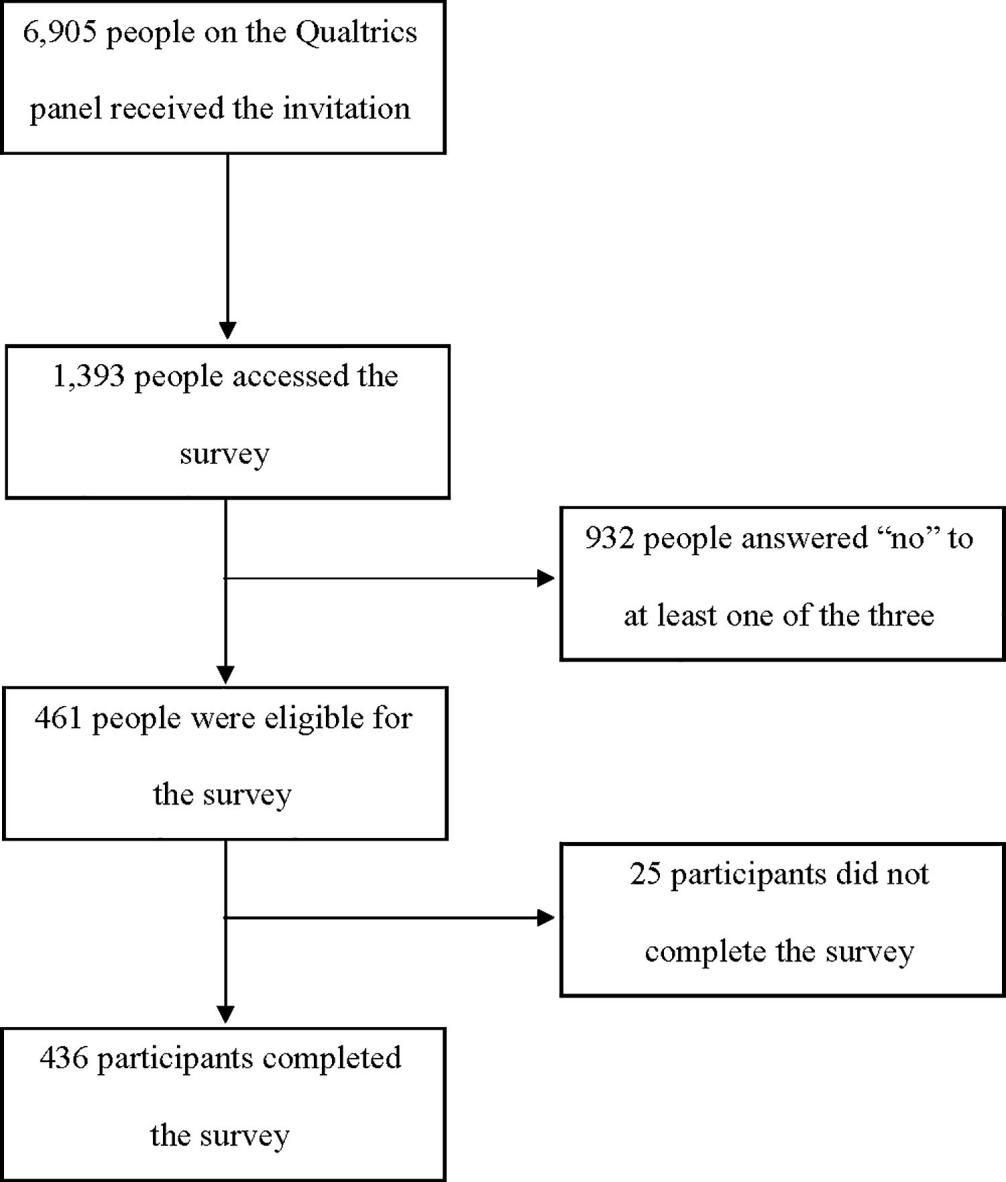
Flow diagram of survey participation.

Approximately 6,905 individuals on the Qualtrics panel received the invitation, of whom 1,393 accessed the survey (20.2%). After giving consent to participate, 932 participants answered “no” to at least one of the three screening questions (including receiving Medicaid) and were excluded (66.9%). Of those who answered “yes” to all three screening questions (n = 461), 25 did not finish the survey (Fig 1). Of those who were eligible (answering “yes” to all screening questions) and consented to participate, 94.6% completed the survey. We analyzed data from 436 participants who met the above eligibility criteria (being a Medicaid enrollee) and completed the survey. The average survey completion time was 15.5 minutes (range 3.9–320.3).

All participants self-reported being Medicaid enrollees. The majority of participants were female (82.3%) and the mean age was 40.5 years. Most participants identified as White or Caucasian (68.8%), followed by Black or African American (19.7%) and Spanish or Latino/a (8.7%). The vast majority (85.8%) did not have a bachelor’s degree, 41.5% reported having a high school diploma or less and 44.3% reported having some college education or an associate degree. See Table 2 for participant characteristics.

**Table 2:** Participant characteristics.

Characteristics	n (%) (unless otherwise specified)
**Gender**	
Female	359 (82.3)
Male	77 (17.7)
**Age**	Mean 40.5 years (SD: 14.8)
	Range 18–78
Race/Ethnicity*	
American Indian or Alaska Native	14 (3.2)
Asian	10 (2.3)
Black of African American	86 (19.7)
Native Hawaiian or Other Pacific Islander	1 (0.23)
White or Caucasian	300 (68.8)
Spanish or Latino/a	38 (8.7)
Other	7 (1.6)
**Education**	
Less than high school diploma	35 (8.0)
High school diploma or equivalent	146 (33.5)
Some college or associate degree	193 (44.3)
Bachelor’s degree or higher	62 (14.2)
Chronic Conditions*	
Arthritis	102 (23.4)
Cancer	18 (4.1)
Chronic obstructive pulmonary disorder	25 (5.7)
Depression or anxiety	182 (41.7)
Diabetes	43 (9.9)
Heart disease	8 (1.8)
Hypertension	91 (20.9)
History of stroke	8 (1.8)
Other	53 (12.2)
**Family history of cancer**	
Yes	260 (59.6)
No	176 (40.4)

*Participants could select more than one.

For brevity, we only report odds ratios and 95% confidence intervals where there was statistical significance. See Table 3 for all analyses.

**Table 3:** Odds ratios and 95% CIs assessing the relationship between sociodemographic characteristics and graph literacy, subjective numeracy, and health literacy^^^.

	High graph literacy (>1)	High subjective numeracy (>11)	High health literacy
High subjective numeracy (>11)	1.49		
	1.02–2.18		
High health literacy	1.33	2.66*	
	0.77–2.28	1.48–4.78	
Age	1.01	1.01	1.02*
	1.00–1.02	1.00–1.03	1.00–1.04
Gender	1.37	0.42*	0.88
	0.83–2.25	0.27–0.70	0.43–1.82
Education			
Less than high school	1 (referrent)	1 (referrent)	1 (referrent)
High school degree or equvalent	1.58	2.12	2.67*
	0.73–3.42	0.95–4.73	1.11–6.43
Some college	2.06	2.14	2.36*
	0.97–4.38	0.97–4.70	1.02–5.44
College or higher	1.92	4.83**	4.56
	0.81–4.52	3.07–19.97	1.41–14.72
Family history of cancer	1.38	1.12	0.51*
	0.94–2.02	0.76–1.64	0.28–0.93
One or more chronic conditions	1.25	1.12	1.10
	0.84–1.87	0.75–1.67	0.62–1.92

^ Top column variables were treated as the outome, each row variable was treated as the exposure. Simple logistic regression used for all analyses.

*p < .05

** p < .001.

### Results corresponding to the primary aim

#### Health literacy and sociodemographic characteristics

A total of 374 participants (85.8%) reported adequate health literacy. There was no statistically significant relationship between health literacy and gender (p = .73) or having at least one chronic condition (p = .75). Respondents with adequate health literacy were more likely to have higher education (OR = 1.40, 95% CI 1.01–1.95, p = .04) and older (OR = 1.02, 95% CI 1.00–1.04, p = .024). Respondents with a family history of cancer had lower odds of adequate health literacy (OR = 0.51, 95% CI: 0.28–0.93, p = .03).

#### Numeracy (SNS3) and sociodemographic characteristics

The mean SNS3 score was 11.1 where a higher score indicated higher subjective numeracy (SD = 4.08, range = 3–18, median = 11). When dichotomized, 48.9% of participants (213/436) were in the high numeracy group and 51.2% (223/436) were in the low numeracy group. The was no statistically significant relationship between SNS3 and a family history of cancer (p = .56) or having chronic conditions (p = .57). Women were less likely than men to have high numeracy (OR = 0.42, 95% CI 0.25–0.70, p < .001). Respondents with a bachelor’s degree or higher had greater odds of high numeracy (OR = 7.83, 95% CI 3.07–19.97, p < .001).

#### Graph literacy and sociodemographic characteristics

The mean graph literacy score was 1.47 (SD 1.05, range 0–4, median 1) where a higher score indicates higher graph literacy. When dichotomized, 52.1% of participants (227/436) were in the low graph literacy group and 47.9% (209/436) were in the high graph literacy group. The relationship between graph literacy and gender (p = .22), having chronic conditions (p = .27), having a family history of cancer (p = .10), or higher education (p = .73) was not statistically significant.

#### Health literacy, subjective numeracy, and graph literacy

In the bivariate analysis, the relationship between graph literacy and health literacy was not statistically significant (p = .31). Respondents with higher SNS3 had higher odds of adequate health literacy (OR = 2.66, 95% CI 1.48–4.78, p < .001). Respondents with higher graph literacy had greater odds of higher SNS3 (OR = 1.49, 95% CI: 1.02–2.18, p = .037). In the multiple logistic regression, graph literacy was not associated with any independent variable.

### Results corresponding to the secondary aim

#### Comprehension, preference, and graph literacy

**Fig 2:**
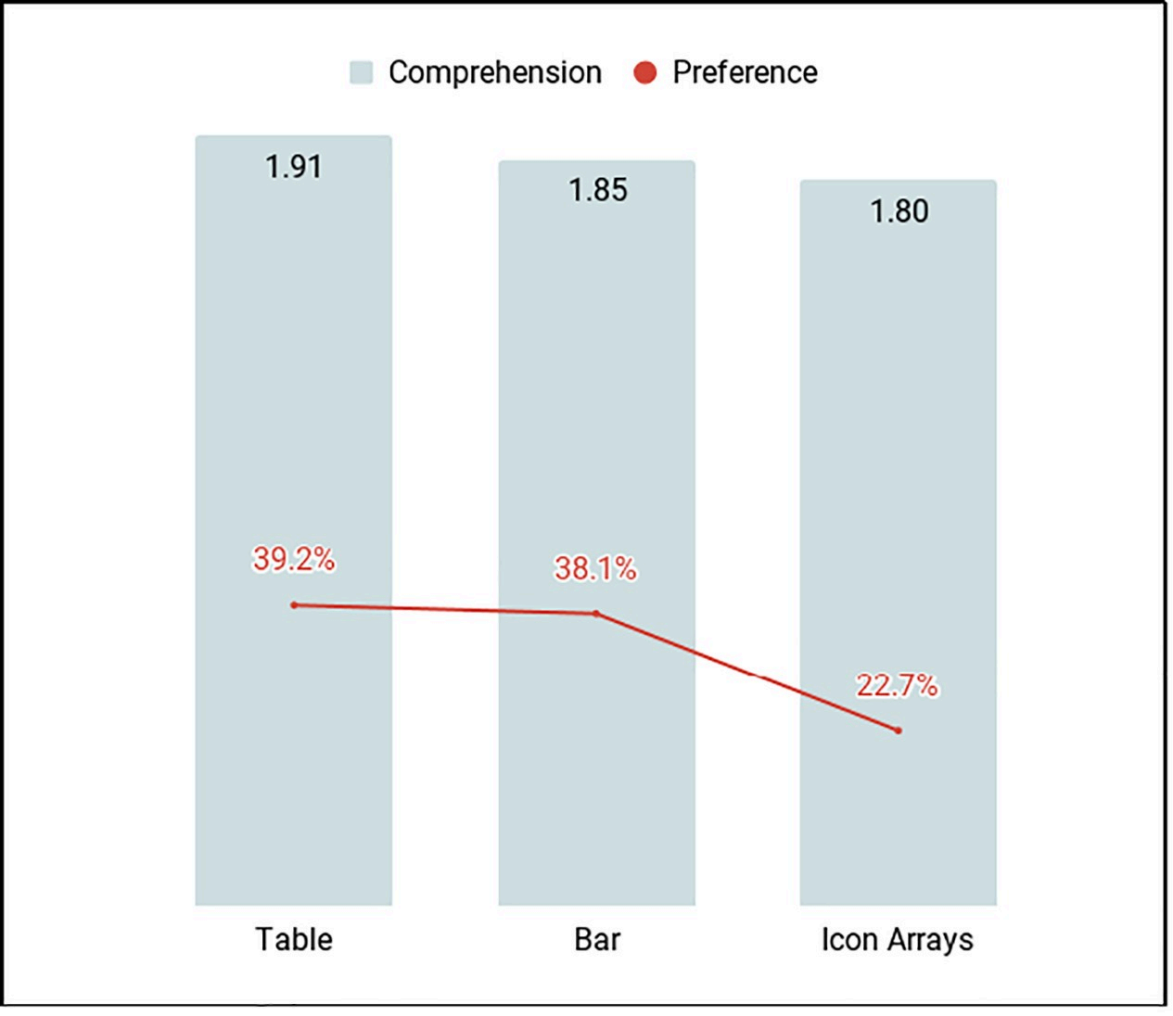
Mean comprehension score (range 0–3) by graphical format and reported preference for one of the three formats.

Mean comprehension score by format was highest for table (1.91), then bar graph (1.85), followed by icon array (1.80) (possible range 0–3, p = .02 when comparing table and icon scores). Mean overall comprehension score was 5.6 (SD = 2.5). The majority of the sample preferred tables or bar graphs (39.2% and 38.1% respectively) (Fig 2).

In the simple logistic regressions, higher overall comprehension was associated with higher graph literacy (OR = 1.31, 95% CI 1.20–1.42, p < .001) and preference for icons was associated with lower graph literacy compared to preference for tables (OR = 0.59, 95% CI 0.35–0.97, p = .04). In the multiple logistic regression controling for preference, SNS3 score, age, education, history of chronic disease, family history of cancer, and health literacy, graph literacy was still strongly associated with overall comprehension (OR = 1.29, 95% CI 1.18–1.41, p < .001).

#### Comprehension and preference

In the simple linear regression, preference for bars or icon arrays was associated with lower overall comprehension scores when compared to preference for tables (bars: coefficient = -0.82, 95% CI -1.35, -0.28, p = 0.003; icon arrays: coefficient = -1.11, 95% CI -1.73, -0.49, p < .001). In the multiple linear regression, preference for bars or icon arrays was still associated with lower overall comprehension scores when compared to preference for tables (bars: coefficient = -0.74, 95% CI -1.25, -0.23, p = .005; icon arrays: coefficient = -0.88, 95% CI -1.48, -0.28).

#### Order effects

When looking at order effects, we found that participants who saw bar graphs or icon arrays first had lower overall comprehension scores when compared to seeing tables first (bar: -0.62, 95% CI -1.20, -0.03, p = .039, icon arrays: -0.68, 95% CI -1.27, -0.09, p = .024). If participants saw icon arrays first they had a lower icon array score than if they saw the tables or bar charts first (OR = 0.76, 95% CI 0.66–0.93, p = .008). No other order effects were significant.

## Discussion

### Summary of main findings

We found a positive relationship between graph literacy and numeracy. Numeracy was related to gender, education, and health literacy. Only graph literacy was significantly associated with the total graph comprehension score. There was alignment between comprehension scores and preferred visual display format, with tables being preferred and yielding the highest comprehension score. Icon arrays yielded the lowest comprehension score and were least preferred. Regarding order effects, participants who saw bar graphs or icon array first had lower overall comprehension scores.

### Strengths and limitations of the study

This study was the first online survey of graph literacy in a Medicaid eligible population with lower education conducted in the US. The characteristics of our sample were broadly representative of the Medicaid enrolled population. A major strength is the inclusion of participants on Medicaid, with lower education and lower graph literacy. Most study limitations are related to the nature of online survey data collection and the highly selected nature of the study sample. The measures used have not been fully validated for online use although most have previously been used with online samples. The response rate was low when considering the large number of people who received the online survey invitation (6,905). However, of those who accessed the survey and were eligible (answering “yes” to all screening questions), 94.6% completed the survey. Our sample was derived from an online panel of respondents (who did not communicate with one another, as far as can be determined) and may not be fully representative of the US population receiving Medicaid, with lower education, and presumed lower SES. Social desirability bias could have affected the responses participants provided, particularly on the subjective health literacy (85.8% of our sample self-reported adequate health literacy) and numeracy scales. This bias may have led some participants to respond to those questions based on social expectations, particularly for participants with higher education levels. The survey response rate of 20.2% is aligned with average online survey responses rates in online panels [38, 39].

### Comparison with other studies

The mean graph literacy score was considerably lower (1.47, SD 1.05) than mean scores reported in the validation of the short form graph literacy scale in a sample of the US (mean: 2.21, SD 1.12) and German populations (mean: 2.03, SD 1.10). This may indicate that people of lower educational attainment and lower SES have considerably lower graph literacy than the general population and may not always benefit from information presented graphically. This hypothesis may also be supported by the fact that 39.2% of our sample reported preferring using the table to process numeric health information, while seemingly understanding information better in this format.

Nayak’s study of graph literacy in a highly educated sample of prostate cancer patients assessed graph literacy using the 13-item version of the scale [22]. It is therefore difficult to compare the full scale mean score to the short version’s score used in our study. However, consistent with our findings, Nayak et al. found that numeracy and graph literacy scores were correlated, suggesting that people with lower numeracy also have lower graph literacy. Brown et al., in a sample with higher educational attainment, also found that less numerate individuals may have less ability to interpret graphs [40, 41]. Our study found a similar relationship between subjective numeracy and graph literacy. Those findings question the previously accepted hypothesis that graphical representation of risks improves understanding in individuals with lower numeracy [6, 14, 42, 43]. Further research is needed.

Mean numeracy scores in our sample of survey respondents with lower educational attainment were considerably lower than numeracy scores found in Nayak’s highly educated sample, and McNaughton’s study [22, 44]. Several studies have shown that education does not predict numeracy [45–47]. In our study population, and consistent with Brown’s finding in a higher SES sample, education was related to subjective numeracy and health literacy [40]. Numeracy was also associated with health literacy (p < .001), consistent with Brown’s finding among higher SES participants.

In our sample, preference for a visual display format coincided with better comprehension. This finding is not consistent with prior studies [15, 40]. In this sample, the table format was preferred, immediately followed by the vertical bar graph. In Brown’s study, where tables had not been introduced, the vertical bar graph was preferred. Regarding comprehension, tables and vertical bar graph were best understood, closely followed by icon arrays. In previous research, both bar charts and icon arrays have been shown to perform best, depending on the type of task at hand (verbatim versus gist), the denominator used etc. [15]. In Scalia’s recent study, patients recruited from a vascular clinic preferred the pie chart format over icon array and reported better realization that risks increase with time for each option [48]. Given the small comprehension score differences between visual display formats but a significant association between stated preference and comprehension score in our sample, further research is required.

### Practice implications

In light of our findings, we may want to investigate those issues further and possibly reconsider the use of graphic display formats among people of lower graph numeracy, who are more likely to hace lower education and lower numeracy. Further research that compares visual display formats among people of lower SES recruited in community settings (to prevent potential biases introduced by online samples) may be warranted. Offering multiple ways to present and process numeric health information, including numbers alone using natural frequencies as well as bar charts (given the stated preference) may be worth exploring [15]. Should clinicians and developers of health information also consider patient preferences in communicating risks? Effective communication strategies should consider the impact of lower education and lower numeracy on graph literacy among patients of lower SES. Alternative strategies that use video messages and nonverbal cues (e.g., voice intonations, facial expressions) as well as words to convey affective and cognitive meaning of the numbers to improve patient comprehension of numeric health information require further investigation [49].

## Conclusions

Despite frequently reporting adequate health literacy, graph literacy among a Medicaid eligible population was considerably lower than the mean scores previously collected in the general population in the US. Our study findings suggest that in people of lower educational attainment, graph literacy seems to be the strongest predictor of graph comprehension and is correlated with numeracy. Further, preference for a particular display format mirrored comprehension on this format. Both tables and bar charts lead to slightly higher comprehension scores than icon arrays, and are thus understood differently by different people. It may therefore be necessary to reconsider the use of graphic display formats when designing information for people with lower educational levels. Given the stated limitations of the present survey, further research in this area is needed.

## Supporting information

S1 File CHERRIES checklist. (PDF)

S2 File Final survey. (PDF)
