# Supplementary material for: Graph literacy matters: Examining the association between graph literacy, health literacy, and numeracy in a Medicaid eligible population
Source: PLoS One. 2020 Nov 11;15(11):e0241844. doi: 10.1371/journal.pone.0241844 (PMC7657552; doi:10.1371/journal.pone.0241844)
Supplement: S2 File — (PDF) [file pone.0241844.s002.pdf]

Information Sheet and Consent

INFORMATION SHEET

Understanding the link between health literacy, numeracy, and graph literacy: an online survey

This research project is being conducted by Professor Marie-Anne Durand from The Dartmouth Institute for Health Policy & Clinical Practice at Dartmouth College, Hanover, NH, USA. It is a study about the association between health literacy, numeracy, and graph literacy. The goal of this study is to identify the best format of medical information to enhance communication with patients. Your participation is voluntary and involves completing a brief survey. You may opt out of the survey at any time.

The information collected will be maintained confidentially. Any personally identifiable information will not be used in any presentation, publication, or other written document about this project.

Questions about this project may be directed to: Professor Marie-Anne Durand at marie-anne.durand@dartmouth.edu or 603-653-0851 during normal business hours.

I consent to participate in this survey.

- ◯ Yes
- ◯ No

I am 18 years of age or older.

- ◯ Yes
- ◯ No

I have Medicaid.

- ◯ Yes
- ◯ No

I am comfortable reading and completing a survey in English.

- ◯ Yes
- ◯ No

Demographic questions

How do you describe yourself?

- ◯ Male
- ◯ Female
- ◯ Other
- ◯ Prefer not to say

What is your age?

[ ]

Which groups do you identify with? Please select all that apply.

- ☐ American Indian or Alaska Native
- ☐ Asian
- ☐ Black or African American
- ☐ Native Hawaiian or Other Pacific Islander
- ☐ White or Caucasian
- ☐ Spanish or Latino/a
- ☐ Other

What is the highest level of school you have completed?

- ○ Less than a high school diploma
- ○ High school diploma or equivalent (e.g., GED)
- ○ Some college or associate degree
- ○ Bachelor's degree or higher

Do you have any of the following chronic conditions? Please select all that apply.

- [ ] Arthritis
- [ ] Cancer
- [ ] Chronic obstructive pulmonary disorder (COPD)
- [ ] Depression or anxiety
- [ ] Diabetes
- [ ] Heart disease
- [ ] Hypertension (High blood pressure)
- [ ] History of stroke
- [ ] ____________ Other
- [ ] None

Do you have a family history of cancer?

Family history refers to having one or more first- or second-degree relative with cancer. First-degree relatives include parents, brothers, sisters, and children. Second-degree relatives include aunts, uncles, nieces, nephews, grandparents, and grandchildren.

- ○ Yes
- ○ No

Health Literacy

How confident are you filling out medical forms by yourself?

- ◯ Extremely
- ◯ Quite a bit
- ◯ Somewhat
- ◯ A little bit
- ◯ Not at all

Graph Literacy

Please review the graphs and answer the questions that follow.

Here is some information about different forms of cancer.

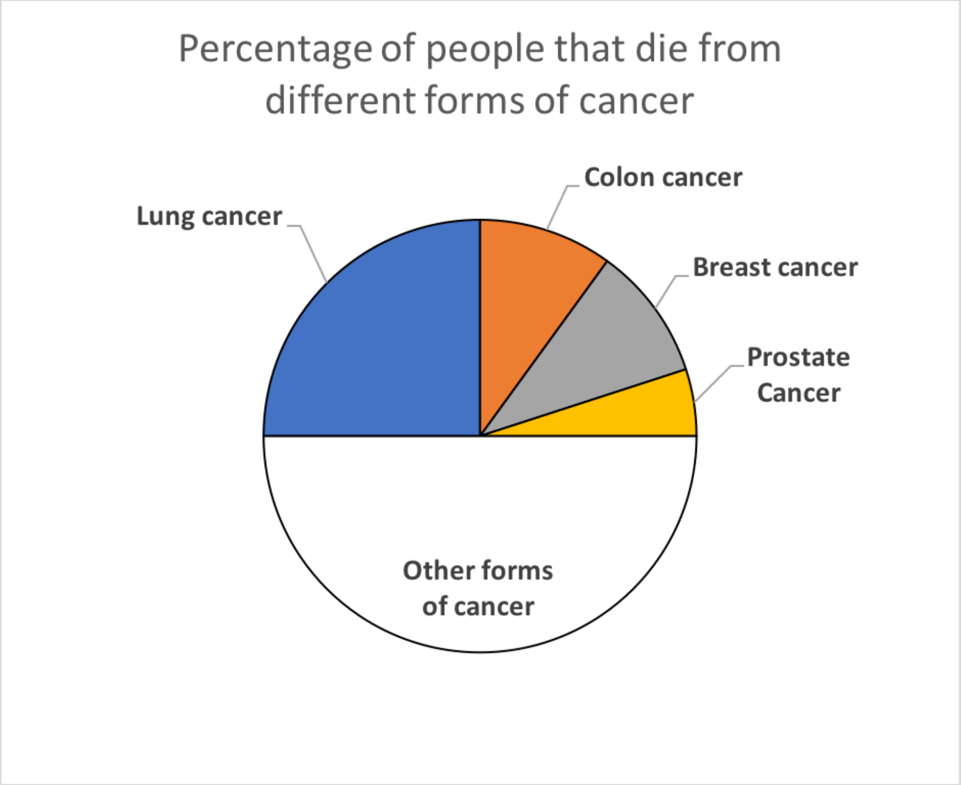

Approximately what percentage of people who die from cancer die from colon cancer, breast cancer, and prostate cancer taken together?

The following figure shows the number of men and women among patients with disease X. The total number of circles is 100.

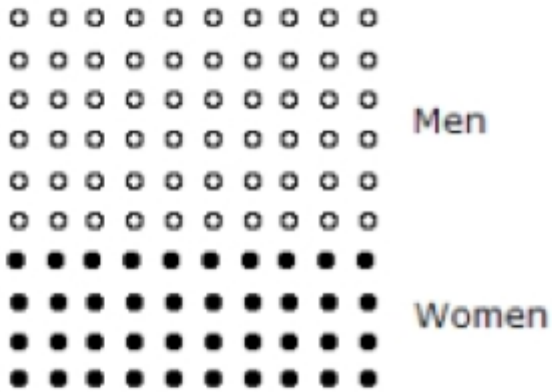

How many more men than women are there among 100 patients with disease X?

In the newspaper you see two advertisements, one on page 15 and another on page 17. Each is for a different treatment of psoriasis, and each includes a graph showing the effectiveness of the treatment over time.

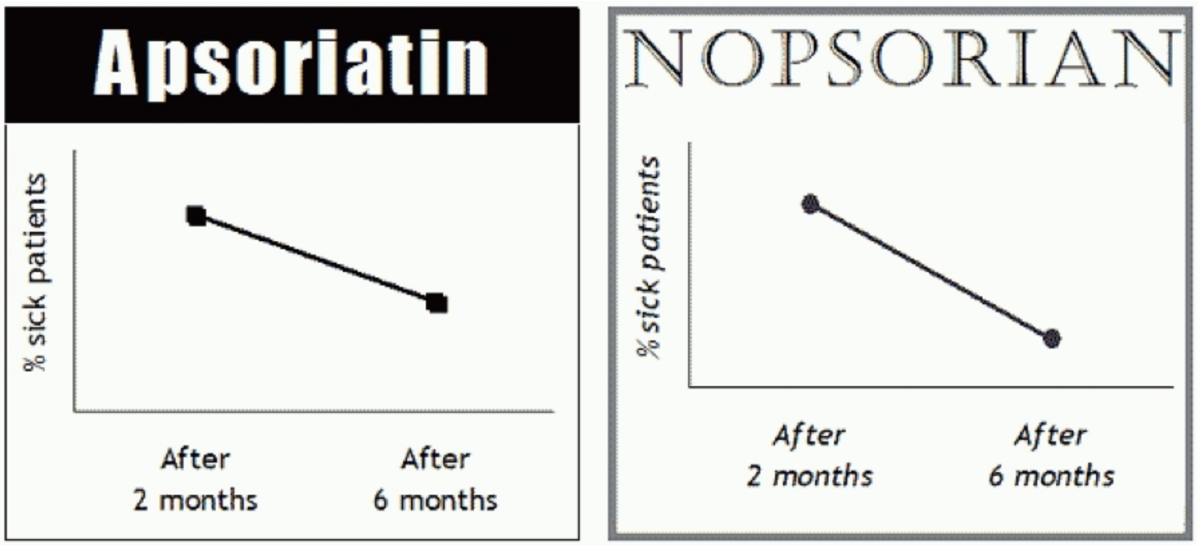

Which of the treatment contributes to a larger decrease in the percentage of sick patients?

- ◯ Apsoriatin
- ◯ Nopsorian
- ◯ They are equal
- ◯ Can't say

In a magazine you see two advertisements, one on page 5 and another on page 12. Each is for a different drug for treating heart disease, and each includes a graph showing the effectiveness of the drug compared to a placebo (sugar pill).

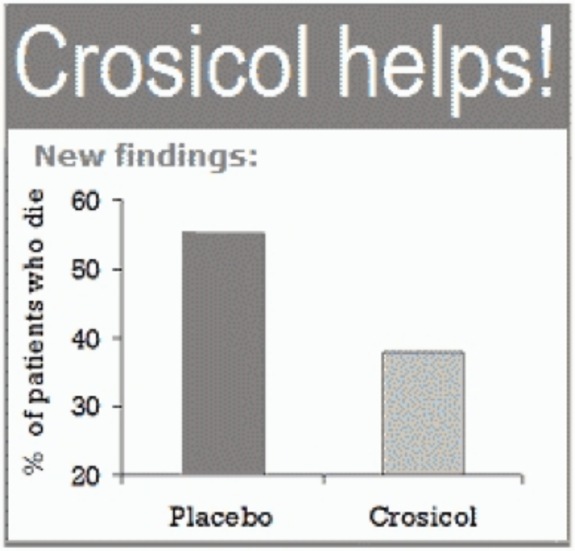

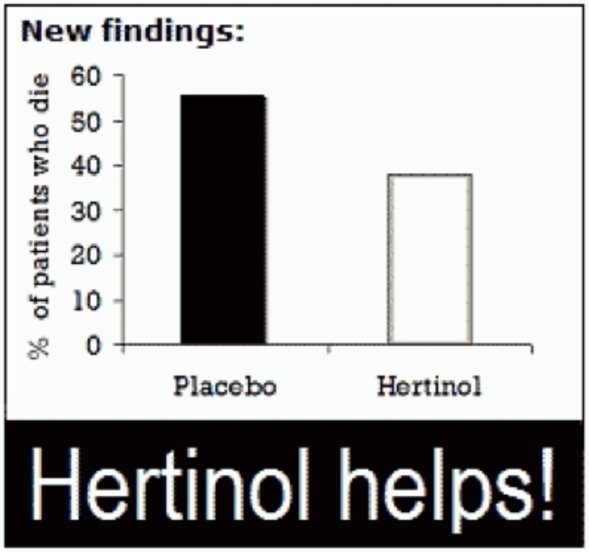

Compared to the placebo, which treatment leads to a larger decrease in the percentage of patients who die?

- Crosicol
- Hertinol
- They are equal
- Can't say

Subjective Numeracy Scale

For each of the following questions, please check the box that **best reflects your answer.**

How good are you at working with fractions?

**Not at all good** 1	2	3	4	5	**Extremely good** 6
○	○	○	○	○	○

How good are you at figuring out how much a shirt will cost if it is 25% off?

**Not at all good** 1	2	3	4	5	**Extremely good** 6
○	○	○	○	○	○

How often do you find numerical information to be useful?

**Never** 1	2	3	4	5	**Very often** 6

○ ○ ○ ○ ○ ○

Block 8

You will now view different charts or graphs. The charts or graphs describe the risk of cancer coming back following different treatments. Please look at each graph and do your best to answer the questions on each page.

Comprehension- table

Below is the risk of cancer coming back in 5 and 10 years following treatment X and treatment Y.

Table

	Risk of cancer coming back in 5 years	Risk of cancer coming back in 10 years
Treatment X	30 in 100 women	35 in 100 women
Treatment Y	40 in 100 women	55 in 100 women

Which treatment option has the higher risk of cancer coming back in 5 years?

○ Treatment X
○ Treatment Y
○ There is no difference between the two treatments

According to the table, how many people out of 100 would have cancer coming back in 10 years after having treatment Y?

Using numbers, how would you describe the **difference** in risk of cancer coming back between treatment X and treatment Y after 10 years?

How helpful was this table for understanding the risk of cancer coming back?

- Not at all helpful
- Somewhat helpful
- Very helpful
- Extremely helpful

How confident are you in your answers using this table?

- Not at all confident
- Somewhat confident
- Very confident
- Extremely confident

Comprehension- bar graph

This graph describes the risk of cancer coming back in 5 and 10 years following treatment A and treatment B.

Bar graph

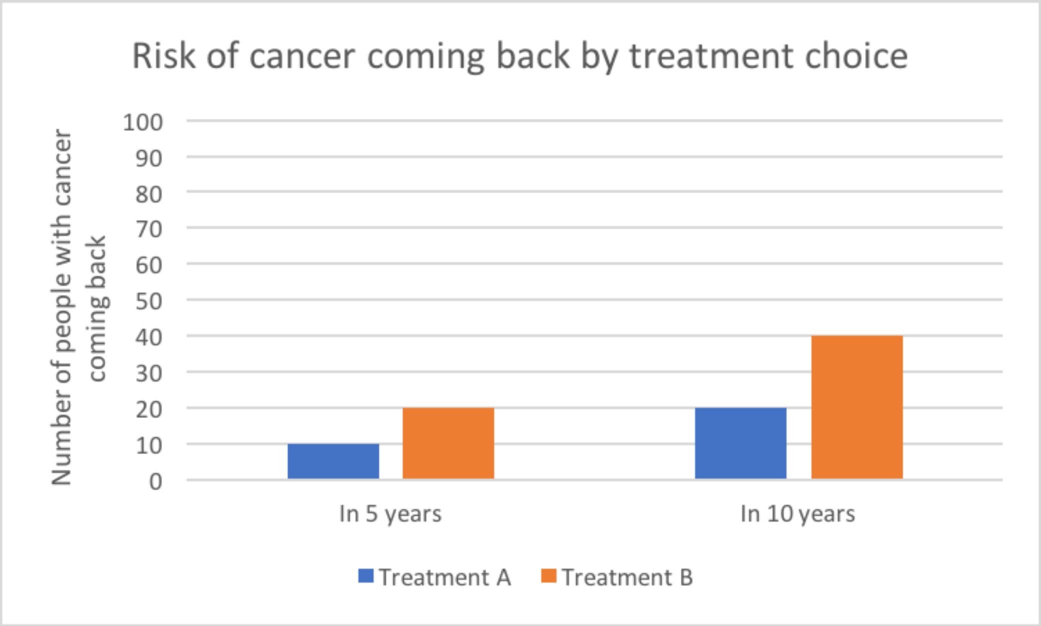

Which treatment option has the least risk of cancer coming back in 5 years?

- Treatment A
- Treatment B
- There is no difference between the two treatments

According to the graph, how many people out of 100 would have cancer coming back in 10 years after having treatment B?

____

Using numbers, how would you describe the **difference** in risk of cancer coming back between treatment A and treatment B after 10 years?

____

How helpful was the bar graph for understanding the risk of cancer coming back?

- ◯ Not at all helpful
- ◯ Somewhat helpful
- ◯ Very helpful
- ◯ Extremely helpful

How confident are you in your answers using this bar graph?

- ◯ Not at all confident
- ◯ Somewhat confident
- ◯ Very confident
- ◯ Extremely confident

Comprehension-icon arrays

Icon arrays

Chance of Cancer Coming Back by Treatment Choice

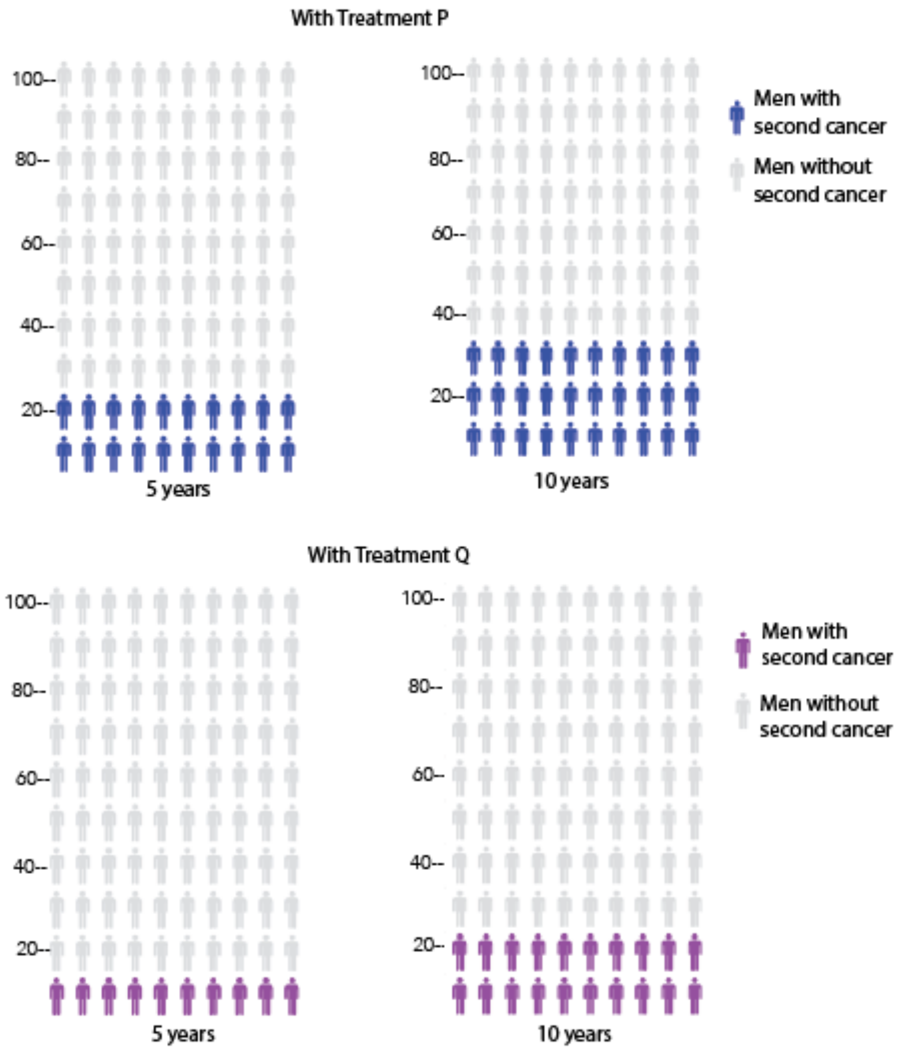

Icon arrays

Chance of Cancer Coming Back by Treatment Choice

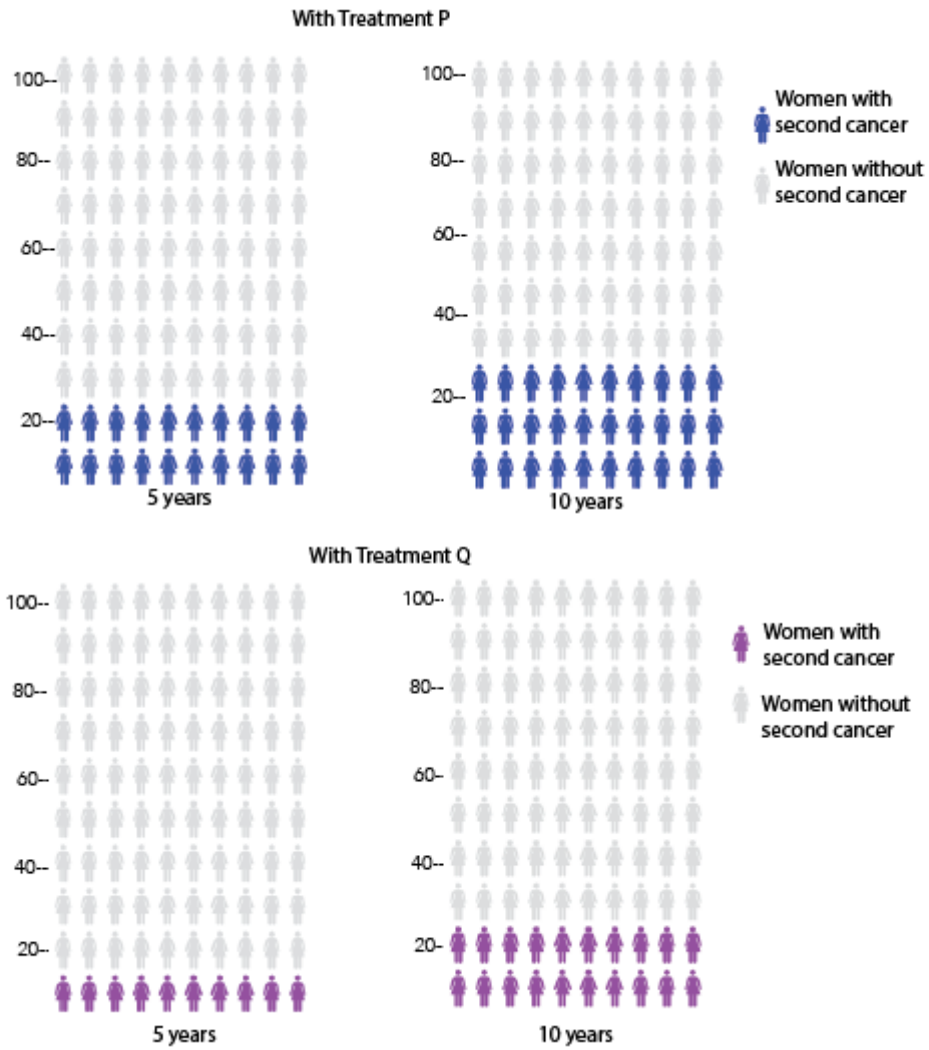

Which treatment option has the least risk of cancer coming back in 5 years?

- ○ Treatment P
- ○ Treatment Q
- ○ There is no difference between the two treatments

According to the icon arrays, how many people out of 100 would have cancer coming back in 10 years after having treatment P?

____

Icon arrays

Chance of Cancer Coming Back by Treatment Choice

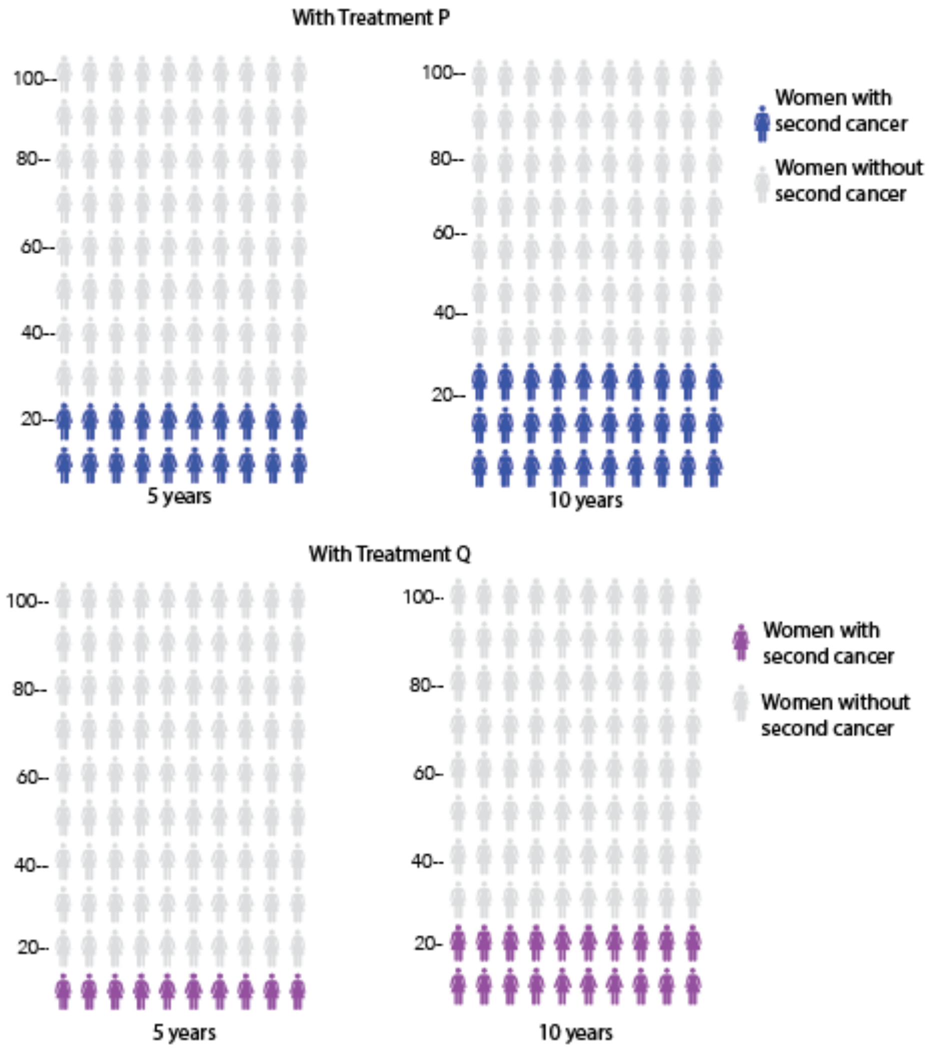

Using numbers, how would you describe the **difference** in risk of cancer coming back between treatment P and treatment Q after 10 years?

How helpful were the icon arrays for understanding the risk of cancer coming back?

- ◯ Not at all helpful
- ◯ Somewhat helpful
- ◯ Very helpful
- ◯ Extremely helpful

How confident are you in your answers using the icon arrays?

- ◯ Not at all confident
- ◯ Somewhat confident
- ◯ Very confident
- ◯ Extremely confident

Preference

Which format did you find most helpful in understanding the risk of cancer coming back?

- ◯ Table

	Risk of cancer coming back in 5 years	Risk of cancer coming back in 10 years
Treatment X	30 in 100 women	35 in 100 women
Treatment Y	40 in 100 women	55 in 100 women

◯ Bar graph

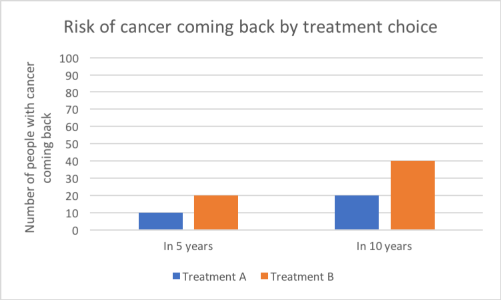

◯ Icon arrays

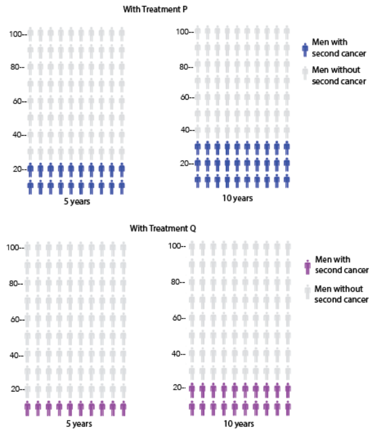

Powered by Qualtrics
